# Feasibility of a fully endoscopic one-anastomosis gastric bypass procedure after endoscopic sleeve gastroplasty in pigs

**DOI:** 10.1016/j.igie.2024.04.002

**Published:** 2024-04-09

**Authors:** Sohaib Ouazzani, Jean-Michel Gonzalez, Loulia Leclercq, Flora Ferrari, Stephane Berdah, Joyce A. Peetermans, Ornela Gjata, Marc Barthet

**Affiliations:** 1 Department of Gastroenterology, Hôpital Erasme, Université Libre de Bruxelles, Brussels, Belgium; 2 Department of Hepatogastroenterology, Hôpital Nord, Assistance Publique-Hôpitaux de Marseille, Aix-Marseille University, Marseille, France; 3 Endo Tools Therapeutics, S.A., Gosselies, Belgium; 4 Department of Digestive Surgery, Hôpital Nord, Assistance Publique-Hôpitaux de Marseille, Aix-Marseille University, Marseille, France; 5 Brussels Medical Device Center (BMDC), Brussels, Belgium; 6 Centre for Surgical Teaching and Research (CERC), Aix-Marseille University, Marseille, France; 7 Endoscopy Division, Boston Scientific Corporation, Marlborough, Massachusetts, USA

## Abstract

**Background and Aims:**

Endoscopic sleeve gastroplasty (ESG) has become an adopted endoscopic gastric volume-reduction procedure, whereas endoscopic gastric bypass is a recent experimental procedure. We assessed the feasibility and safety of combining ESG with a recently developed fully endoscopic one-anastomosis gastric bypass (OAGB) procedure.

**Methods:**

This was a 14-week prospective follow-up of 4 growing pigs using a dedicated gastrojejunal lumen-apposing metal stent (GJ-LAMS) to create a gastrojejunal anastomosis and a duodenal exclusion device (DED). ESG at baseline was followed by the OAGB procedure at weeks 8 to 10 and necropsy at week 14.

**Results:**

In all 4 pigs, endoscopic gastroenterostomy realizing OAGB was possible after ESG. In 1 pig, all study procedures from day 0 to week 14 occurred as intended, with normal health and normal necropsy observations, confirming the feasibility of endoscopic OAGB after ESG. GJ-LAMS migration without DED placement occurred in a second pig. A third pig had an asymptomatic unintended gastrocolic anastomosis discovered at necropsy, and the fourth pig died on day 62 (6 days after GJ-LAMS placement) with the finding of ischemic volvulus on necropsy. At 14 weeks, all remaining stents were removed endoscopically uneventfully. Necropsy was then performed, showing limb lengths ranging from 110 cm to 170 cm, with no inflammation or leaks.

**Conclusions:**

ESG followed by an endoscopic OAGB procedure with a controlled bypass length was technically feasible. One of 4 test animals died shortly after GJ-LAMS placement and before duodenal exclusion.

Gastric endoscopic bariatric treatments (EBTs) include endoscopic sleeve gastroplasty (ESG), which is similar to the most common bariatric surgery currently performed, sleeve gastrectomy.^1^^,^^2^ As the obesity pandemic grows, innovative therapeutic solutions would be beneficial for patients.

We developed in experimental animal studies a fully endoscopic one-anastomosis gastric bypass (OAGB) procedure with duodenal exclusion performed using natural orifice transluminal endoscopic surgery (NOTES) gastrojejunostomy as a potential new EBT.^3^^,^^4^ Our studies suggested that the baseline endoscopic procedure^4^ and conversion to a surgical one-anastomosis duodenal–jejunal bypass^5^ may be feasible in juvenile pigs. Another procedure developed by another research team used a gastrojejunal anastomosis (GJA) and pyloric closure preceded by ESG for gastric volume reduction.^6^ Combining ESG and endoscopic OAGB could offer a new EBT. We tested the feasibility and safety of ESG followed by our endoscopic OAGB procedure in 4 juvenile pigs, with 14 weeks of longitudinal postprocedural follow-up.

## Methods

### Study design

**Figure 1:**
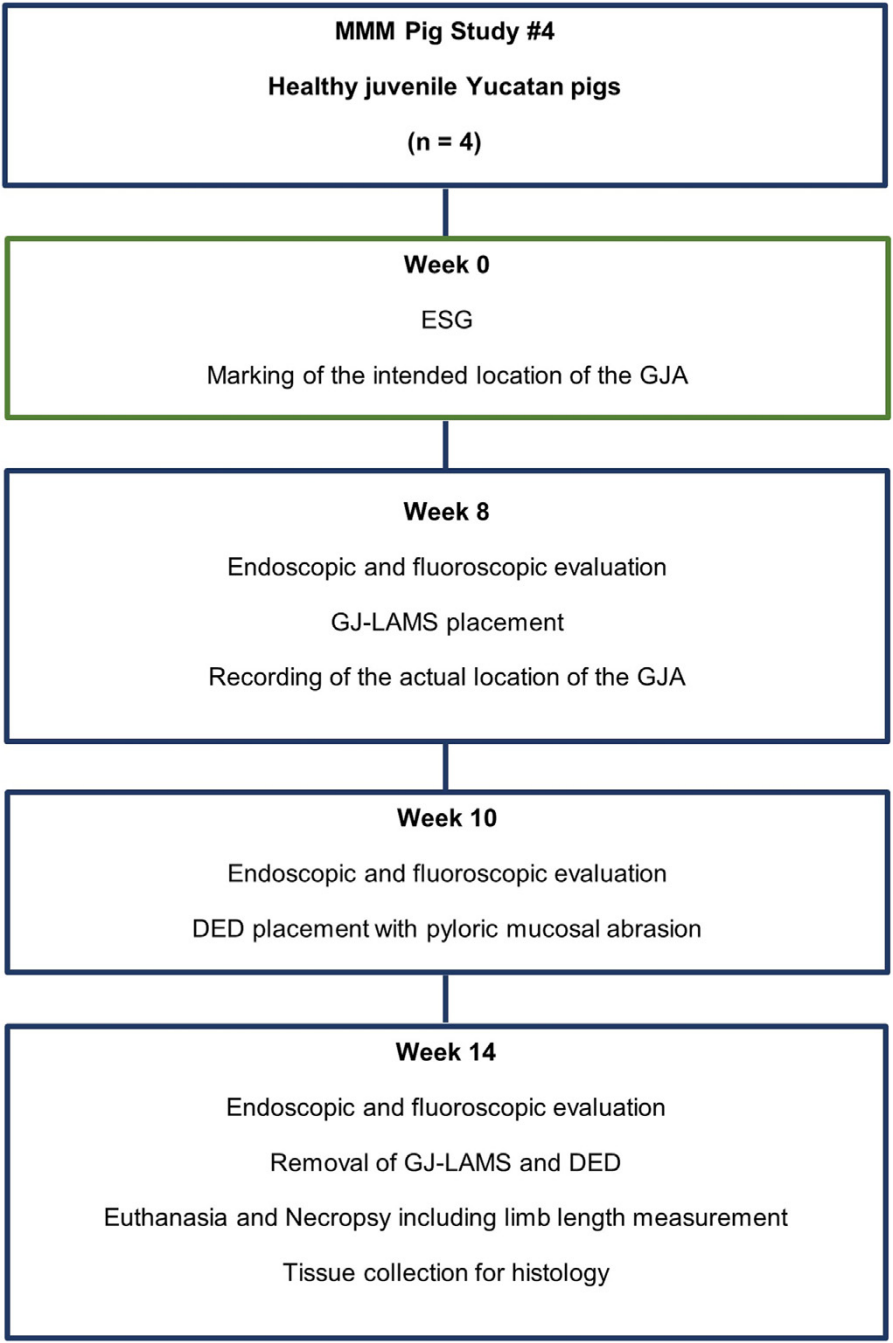
Study flowchart. *MMM*, Marseille, Marlborough, Metabolic; *ESG*, endoscopic sleeve gastrectomy; *GJA*, gastrojejunal anastomosis; *GJ-LAMS*, gastrojejunal lumen-apposing metal stent; *DED*, duodenal exclusion device.

This 14-week prospective animal study was conducted at the Centre for Surgical Teaching and Research, Aix-Marseille University, Marseille, France between July 6 and October 12, 2022 as part of the MMM (Marseille, Marlborough, Metabolic) research program (Fig. 1, Table 1). The Ministère de L'enseignement Supérieur, de La Recherche et de L'innovation (Ministry of Higher Education, Research and Innovation, Paris, France) granted ethical approval for the study. All study procedures followed institutional and national guidelines and were conducted under approval of the Institutional Animal Care and Use Committee and the ethical principles of the Canadian Council of Animal Care. Expert endoscopists at Aix-Marseille University conducted all study procedures and study follow-up.

**Table 1:** MMM (Marseille, Marlborough, Metabolic) preclinical studies

Study	Dates	Intervention	Pig model	No. of experimental pigs	No. of control pigs	Follow-up (wk)	Duodenal exclusion device design
MMM 1^4^	October 2019 to July 2020	First fully endoscopic “MMM” GJ-LAMS and duodenal exclusion device placement in juvenile pigs	Juvenile, normal	7	N/A	38	Asymmetric, partially coated “wine glass” shape. Extended duodenal flange; gastric flange similar to GJ-LAMS flanges.
MMM 2^5^	February 2021 to April 2021	MMM converted to one-anastomosis duodenal–jejunal bypass or sleeve gastrectomy	Juvenile, normal	4	3	12	
MMM 3^8^	May 2021 to August 2021	MMM procedure in obese adult pigs	Adult, obese males	6	2	14	Asymmetric, partially coated “dumbbell” shape. Outer diameter of gastric 1.5 mm larger than duodenal flange.
MMM 4	July 2022 to October 2022	MMM procedure after endoscopic sleeve gastroplasty	Juvenile, normal	4	N/A	14 (MMM procedure on wk 8)	Symmetric, fully uncoated “dumbbell” shape. Duodenal and gastric flanges identical in size.
Total	October 2019 to October 2022		Various models	21	5	12-38	

*GJ-LAMS*, Gastrojejunal lumen-apposing metal stent; *N/A*, not applicable.

The study animals were 4 juvenile Landrace white domestic pigs. Animal handling, preprocedural care, and anesthesia methods were reported previously.^4^^,^^5^

### Endoscopic sleeve gastroplasty

The procedure was performed using the endomina system suturing device (Endo Tools Therapeutics S.A., Gosselies, Belgium) according to the methods described by Huberty et al. ^7^ The procedure was done with 3 or 4 suturing points followed by tattooing and clipping to mark the desired location for subsequent placement of a gastrojejunal lumen-apposing metal stent (GJ-LAMS).

### Fully endoscopic OAGB procedure

**Supplementary Figure 1:**
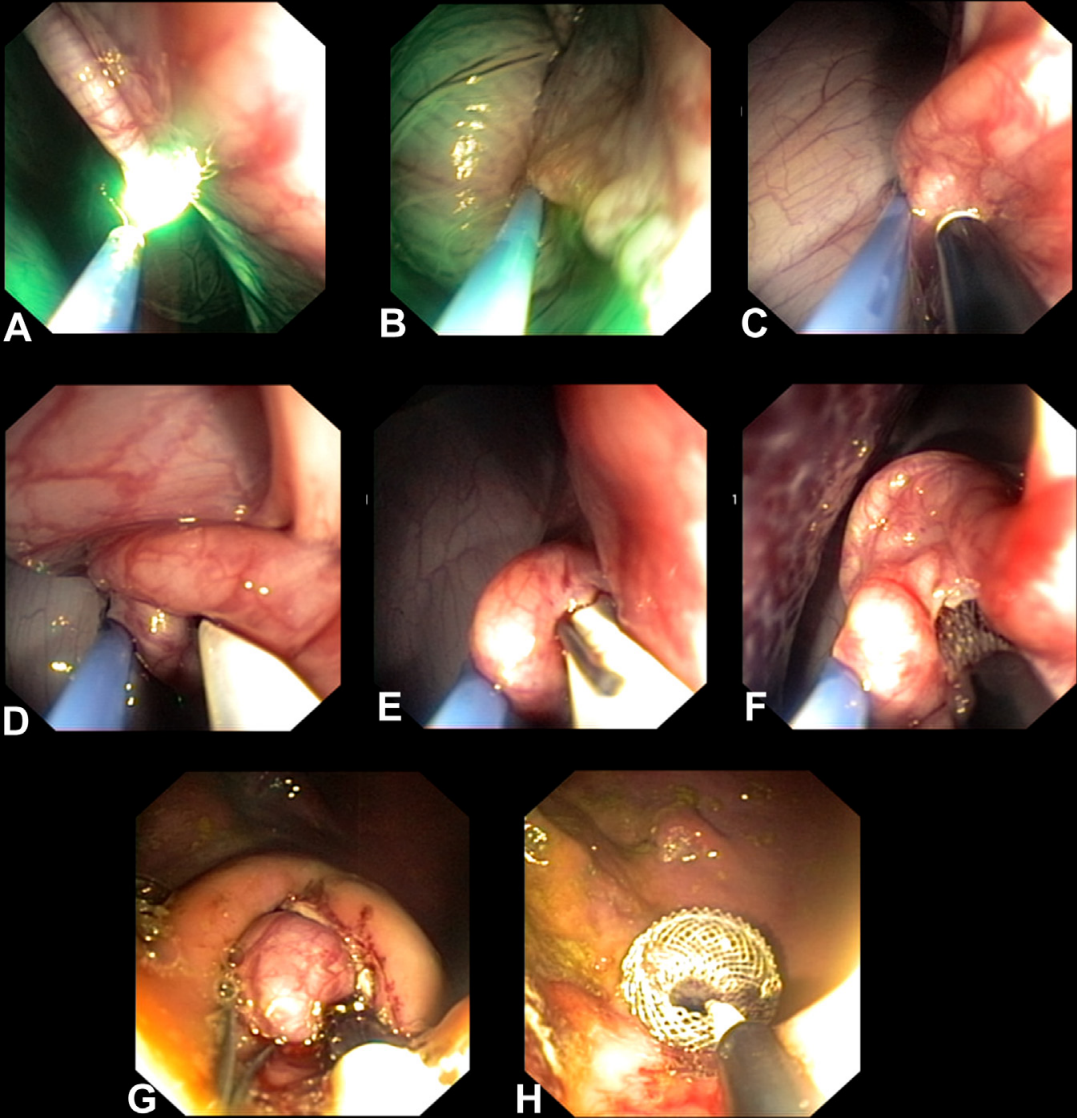
Creation of a gastrojejunal anastomosis at week 0. **A,** A grasper catches the beacon. **B,** The beacon is pushed forward. **C,** The gastrojejunal lumen-apposing metal stent (GJ-LAMS) is cauterized. **D** and **E,** The beacon is pulled back. **F,** The distal flange is deployed. **G,** The grasper is released. **H,** The proximal GJ-LAMS is deployed.

**Supplementary Figure 2:**
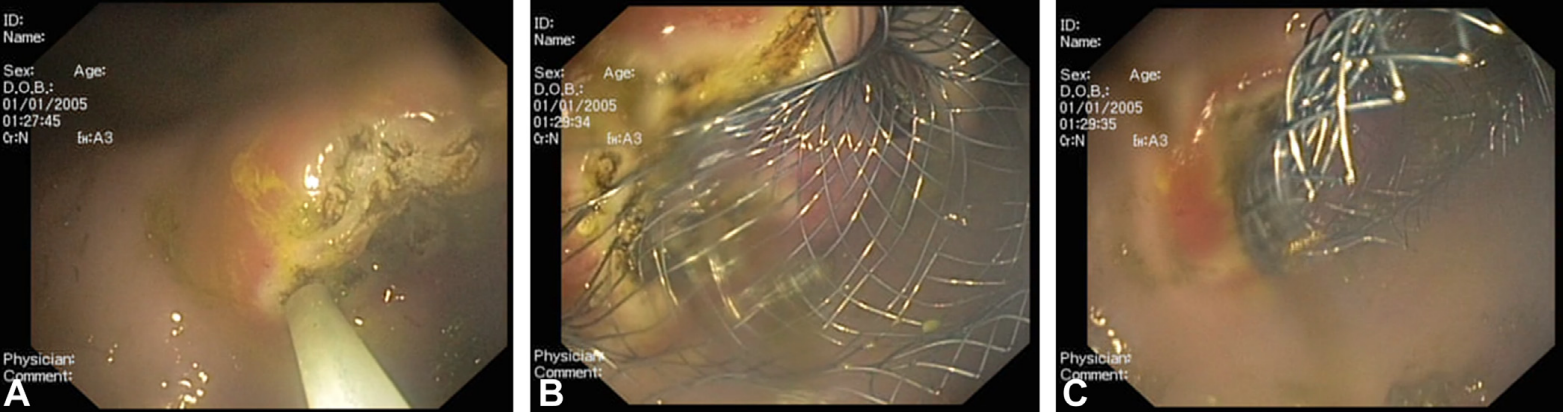
Placement of the duodenal exclusion device (DED) at week 2. **A,** Ablation of the torus pyloricus is performed with an Erbe ICC200 electrosurgical unit (settings: effect 2, cut 160, coagulation 60) (ERBE, Tübingen, Germany). **B** and **C,** The DED is inserted through the scope and deployed.

The NOTES endoscopic OAGB procedure with controlled limb length was performed as reported previously.^4^^,^^5^^,^^8^ Four devices were used: an enteral light beacon, atraumatic grasper, modified 20-mm dedicated GJ-LAMS, and a duodenal exclusion device (DED) (Boston Scientific Corporation [Marlborough, Mass, USA] and Brussels Medical Device Center [Brussels, Belgium]). The procedure was as follows: at week 0, endoscopic measurement of the bypassed limb by jejunal insertion of the light beacon (150-cm target length), followed by a NOTES-based creation of a GJA using a GJ-LAMS (Supplementary Fig. 1, available online at www.igiejournal.org), and at week 2, pyloric occlusion with a DED (Supplementary Fig. 2, available online at www.igiejournal.org).

### Final endoscopic examination, death, and necropsy examination

Fourteen weeks after GJ-LAMS placement, euthanasia was performed using pentobarbital 3 g for each animal under general anesthesia, and necropsy was performed.

### Endpoints

Endpoints were completion of the endoscopic OAGB procedure after ESG (confirmed at necropsy), device-related or procedure-related adverse events, bypass limb length, and gross examination findings at necropsy.

### Statistical analysis

Descriptive statistics were tabulated for continuous variables (weight and procedure duration). The weight change between baseline and week 14 was calculated.

## Results

### Baseline characteristics

All 4 pigs were female, with a mean age of 2.9 ± .1 months at baseline.

### Completion of the ESG procedure

The ESG procedure was uneventful in all 4 pigs on day 0, with pigs receiving 3 (Pig Green and Pig Black) or 4 (Pig Red and Pig Blue) sutures. The mean procedure duration was 68.5 ± 29.7 minutes (range, 46.0-110.0). On week 8, the endoscopic examination revealed a retained gastroplasty in all animals with an overall retention of 71.4% of sutures.

### Completion of endoscopic OAGB procedures

In all 4 pigs, GJ-LAMS placement was successful, although advancement of the light beacon in Pig Blue was difficult with suboptimal visualization under NOTES of the GJ-LAMS placement. The mean total GJ-LAMS placement time was 44.5 ± 2.5 minutes (range, 42.0-48.0).

On week 10, the placement of an endoscopic DED was planned in 3 surviving animals. In Pig Blue, the GJ-LAMS had migrated; thus, no DED was placed. Pig Green and Pig Red underwent successful DED placement, with a mean placement time of 13.7 ± 10.0 minutes (range, 6.0-25.0).

### Animal weight change from baseline

By week 14, the 3 surviving animals had a mean body weight of 49.4 ± 5.6 kg with a mean weight gain of 20.3 ± 4.8 (69.7% ± 16.9% mean gain vs baseline/preprocedural).

### Change in symptoms

Pig Black started to eat less on day 61 and then died on day 62.

### Necropsy examination

**Figure 2:**
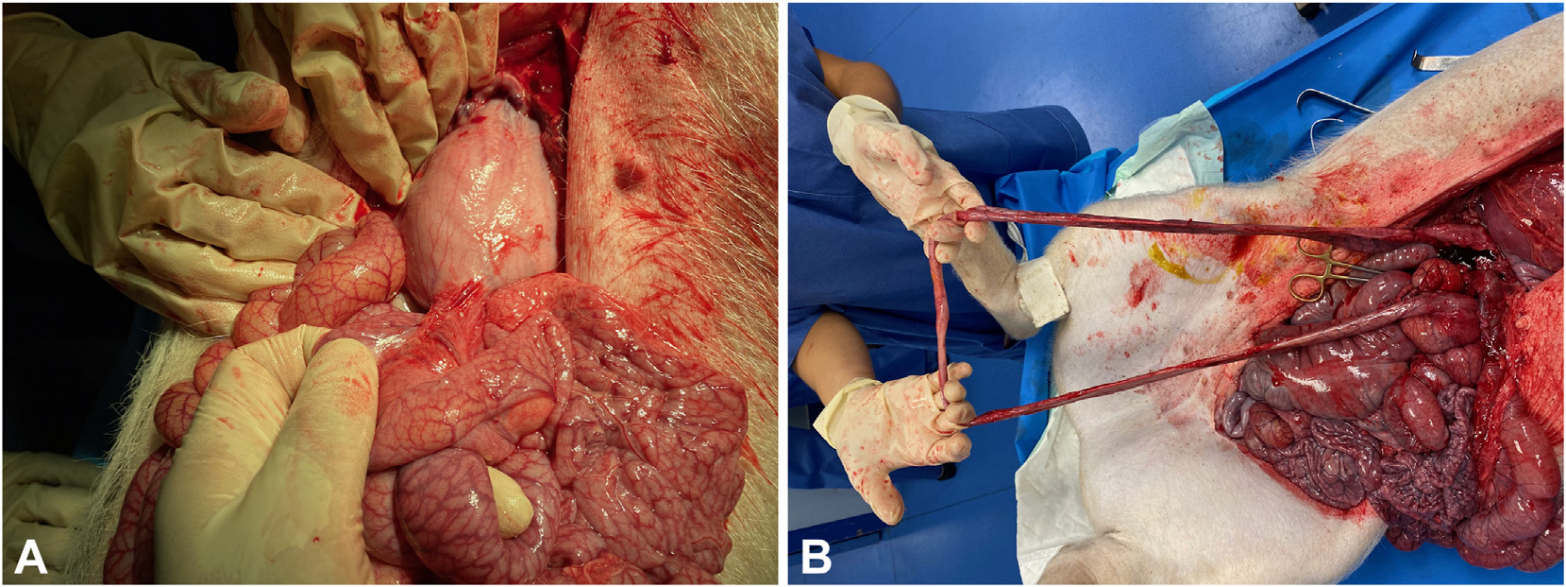
Necropsy specimens showing a gastrojejunal anastomosis (**A**) and bypass limb (**B**).

No inflammation, leakage, or adherence was seen (Fig. 2). In Pig Blue, a migrated GJ-LAMS was found lodged in the jejunal lumen without obstruction. The GJ-LAMS remained in place in Pig Green and Pig Red, but the placement was found to be gastrocolic in Pig Red. The bypass limb length was 170 cm in Pig Green, 140 cm in Pig Red (close to the colonic anastomosis), and 110 cm in Pig Blue.

### Device-related events

**Figure 3:**
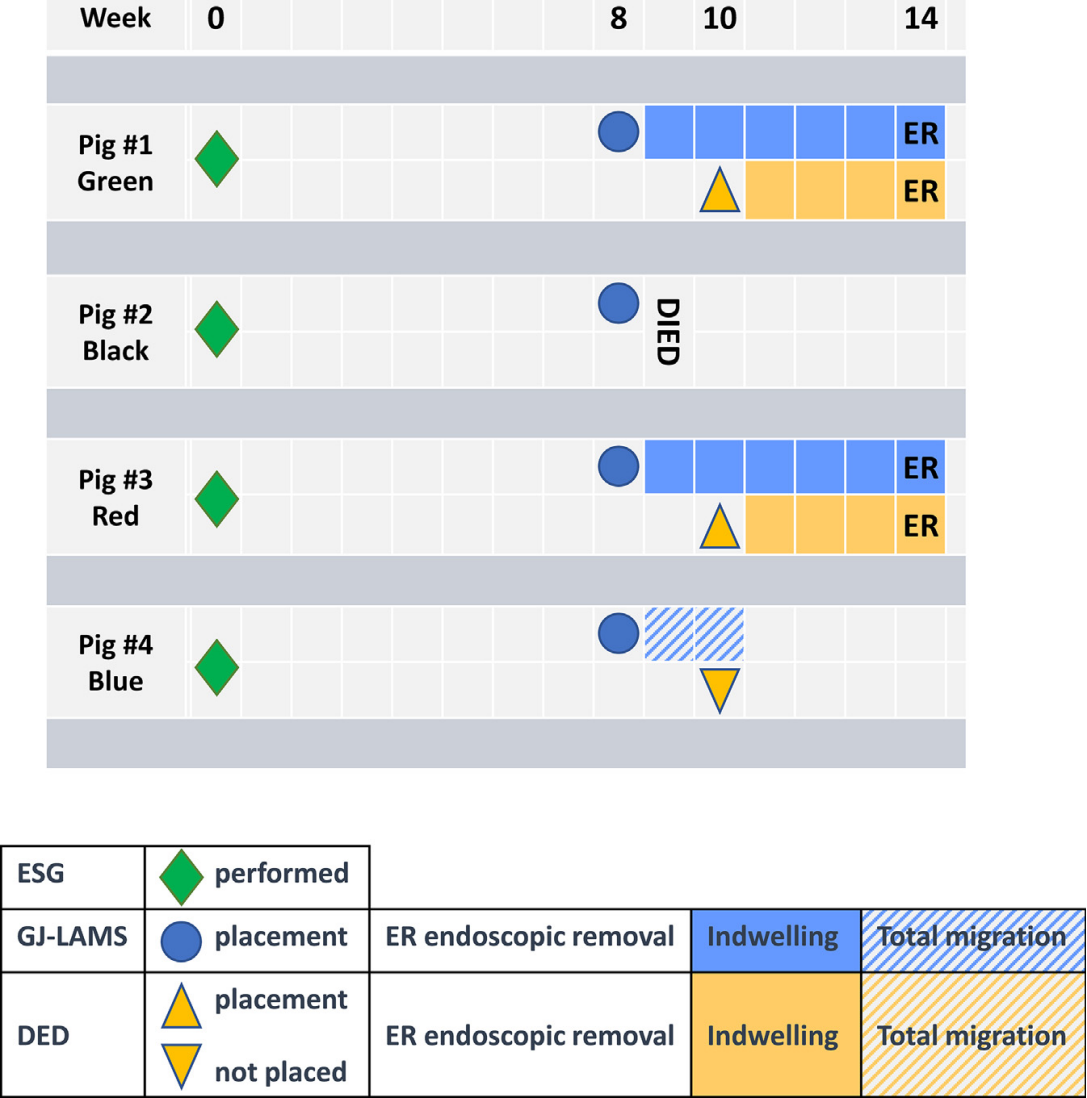
Placement, removal, and migration of GJ-LAMS and DED in adult domestic pigs. *ER,* Endoscopic removal; *ESG*, endoscopic sleeve gastrectomy; *GJ-LAMS*, gastrojejunal lumen-apposing metal stent; *DED*, duodenal exclusion device

GJ-LAMS and DED migrations are documented in Figure 3. Pig Blue had an uneventful stent migration with the GJ-LAMS found in the jejunal lumen and no DED placement.

### Deaths

**Supplementary Figure 3:**
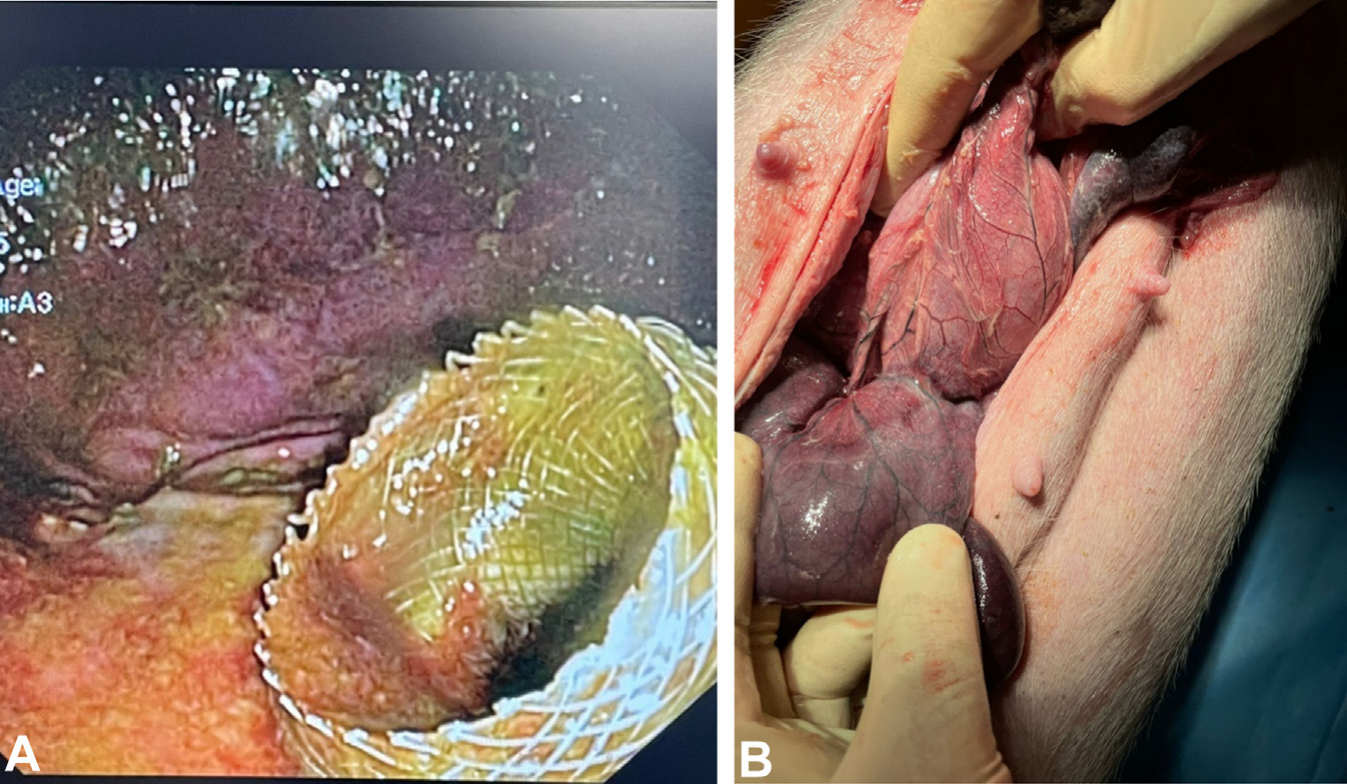
Endoscopic (**A**) and necropsy (**B**) findings of volvulus after Pig Black died on day 62, 6 days after placement of a gastrojejunal lumen-apposing metal stent.

Pig Black died on day 62, 6 days after placement of the GJ-LAMS (Supplementary Fig. 3, available online at www.igiejournal.org). The postmortem upper endoscopy showed the GJ-LAMS in place with a normal-appearing GJA, but gastric stasis was present with pooling of approximately 6 L of fluid. The necropsy revealed small-bowel ischemia and volvulus, without evidence of peritonitis or leakage.

## Discussion

This 14-week pilot study in a juvenile porcine model tested sequential ESG followed by a fully endoscopic NOTES-based bypass bariatric procedure. After ESG, NOTES gastroenterostomies were performed in all pigs. One pig died with the finding of volvulus on necropsy.

The combination of ESG and endoscopic OAGB could potentially improve personalized endoscopic management of obesity and associated metabolic adverse events, with inclusion of gastric volume reduction, controlled bypass limb length, or duodenal exclusion tailored to the patient’s needs.^5^^,^^6^ Our series of pilot studies has had mixed success during longitudinal follow-up after completion of the NOTES-based endoscopic OAGB procedure. At least 1 animal death occurred in 3 of 4 studies (including the current study),^4^^,^^8^ whereas the conversion to a one-anastomosis duodenal–jejunal bypass or sleeve gastrectomy was successful and without pig deaths in a juvenile porcine model.^5^ The current study reinforces the importance of longitudinal follow-up in experimental EBTs. A high level of technical success was achieved in the index procedures. Postprocedural follow-up as long as 37 weeks revealed frequent device migrations that decreased (especially in DED) after procedural improvements and some pig deaths from unknown causes. The current study included a case of fatal volvulus. Because volvulus has been reported after bariatric surgery in case reports,^9^, ^10^, ^11^, ^12^ this might represent a risk of this specific procedure or of bariatric procedures in general.

Our studies had strengths and limitations. We tested a combination of ESG and an EBT previously investigated by at least 1 other research team.^6^ The longitudinal follow-up we included in every study revealed important safety concerns. The gastrocolic anastomosis is a documented type of GJ-LAMS misdeployment.^13^ Because this study included growing pigs without a control group, the intervention’s effect on weight could not be assessed. In conclusion, ESG followed by the planned NOTES-based endoscopic OAGB procedure with duodenal exclusion was completed in 1 of 4 juvenile pigs, with an early GJ-LAMS migration, gastrocolic anastomosis, or death in the other 3.

## Data Sharing

The data that support the findings of this study are available from Boston Scientific Corporation, but restrictions apply to the availability of these data, which were used under license for the current study and so are not publicly available. However, data are available from Professor Marc Barthet (Marc.BARTHET@ap-hm.fr) on reasonable request and with permission of Boston Scientific Corporation.

## Disclosure

The following authors received research support for this study from Boston Scientific Corporation: J.-M. Gonzalez, M. Barthet. In addition, the following authors disclosed financial relationships: L. Leclercq: Full-time employee of Endo Tools Therapeutics S.A. F. Ferrari: Full-time employee of Brussels Medical Device Center, which received a cooperative engineering grant from Boston Scientific Corporation. J. A. Peetermans, O. Gjata: Full-time employee of Boston Scientific Corporation. S. Ouazzani is supported by a research grant from the “Fonds Erasme pour la recherche médicale” (doctoral research fellow grant). All other authors disclosed no financial relationships.
